# Formulation and Bioequivalence of Two Valsartan Tablets After a Single Oral Administration

**DOI:** 10.3797/scipharm.1009-01

**Published:** 2010-11-20

**Authors:** Abdel Naser Zaid, Rita Cortesi, Aiman Qaddomi, Saed Khammash

**Affiliations:** 1 College of Pharmacy, An-Najah National University, P.O. Box: 7, Nablus, Palestine; 2 Department of Pharmaceutical Sciences, University of Ferrara, ViaFossato di Mortara, 17–19, 44100. Ferrara, Italy; 3 Pharmacare Ltd. Beitunia, P.O. Box: 677, Ramallah, Palestine

**Keywords:** Valsartan, Formulation, Bioequivalence, Immediate Release, HPLC, Diovan^®^, Valzan^®^

## Abstract

The aim of this study is to assess the quality of Valzan^®^ tablet (160 mg, valsartan immediate release test formulation) by comparing its pharmacokinetic parameters with Diovan^®^ tablet (160 mg, valsartan reference formulation). Valzan^®^ tablets were prepared according to a dry granulation method (roll compaction). To assess the bioequivalence of Valzan^®^ tablets a randomized, two-way, crossover, bioequivalence study was performed in 24 healthy male volunteers. The selected volunteers were divided into two groups of 12 subjects. One group was treated with the reference formulation (Diovan^®^) and the other one with the generic Valzan^®^, with a cross-over after the drug washout period of 14 days. Blood samples were collected at fixed time intervals and valsartan concentrations were determined by a validated HPLC assay method. The pharmacokinetic parameters AUC_0–48_, AUC_0–∞_, C_max_, T_max_, K_e_ and T_1/2_ were determined for both the tablets and were compared statistically to evaluate the bioequivalence between the two brands of valsartan, using the statistical model recommended by the FDA. The analysis of variance (ANOVA) did not show any significant difference between the two formulations and 90% confidence intervals (CI) fell within the acceptable range for bioequivalence. Based on this statistical evaluation it was concluded that the test tablets (Valzan^®^) is well formulated, since it exhibits pharmacokinetic profile comparable to the reference brand Diovan^®^.

## Introduction

**Fig. 1.**
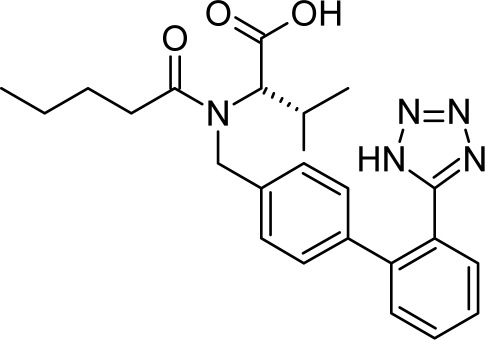
Chemical structure of Valsartan.

Valsartan is chemically *N*-pentanoyl-*N*-{[2′-(1*H*-tetrazol-5-yl)biphenyl-4-yl]methyl}-L-valine (Figure 1).

Valsartan is a nonpeptide, orally active and specific angiotensin II antagonist acting on the AT_1_ receptor subtype present in many tissues, such as vascular smooth muscle and the adrenal gland [1]. Placebo-controlled trials have found valsartan to be both safe and effective for the treatment of hypertension [2]. With valsartan taken in a dosage of 80 to 320 mg once daily, the mean reduction in diastolic blood pressure is 6 to 9 mm Hg, and the mean reduction in systolic pressure is 3 to 6 mm Hg [1]. Studies have shown that valsartan is as effective as enalapril, lisinopril and amlodipine in the treatment of mild to moderate hypertension [3–5]. Valsartan is rapidly absorbed from the gastrointestinal tract after oral administration and can be administered without regard to food intake [6]. The peak effect of valsartan is evident in 2–4 h; the bioavailability is 25%. Valsartan has a half-life of 6–9 h and demonstrates antihypertensive effects for approximately 24 h. Less than 10% of an orally administered dose of valsartan undergoes biotransformation in the liver; the enzymes responsible for its metabolism are unknown, and no active metabolites have been identified [7, 8]. Elimination occurs primarily in the bile (86%) and to a lesser extent via the kidneys (13%), largely as unchanged drug [5, 9]. This study aims to assess the quality of a new generic Valzan^®^ (160 mg valsartan tablets) with the original brand Diovan^®^ (160mg valsartan tablets) by comparing their pharmacokinetic parameters according to the FDA guidelines. Furthermore, the excipients and formulation method used to prepare Diovan^®^ tablets are reported in order to help future studies related to the bioclassification of valsartan.

## Results and Discussion

### Results of validation procedures

**Fig. 2.**
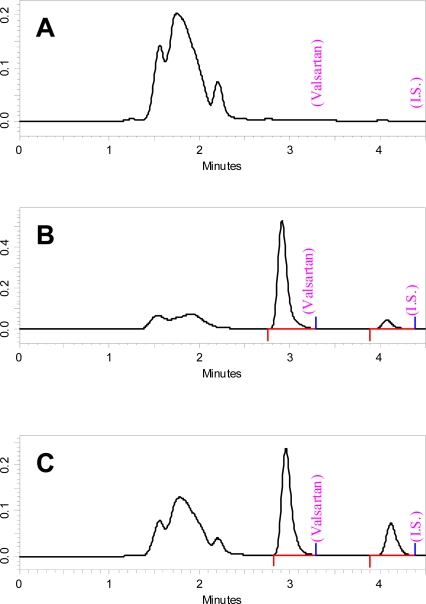
Representative chromatograms for blank plasma sample (A), blank plasma spiked with valsartan to produce drugs concentration of 5 μg/mL and internal standard (B), and plasma samples that was obtained 6 h after administration of the test drug to volunteer # 5 T(C).

Under the chromatographic conditions described, valsartan and the internal standard peaks were completely resolved during the run time of the assay with retention times of 2.9 min and 4.15 min respectively (Figure 2). The broad peak from 1.5–2.3 min is related to plasma components that their concentration may be influenced by the presence of valsartan [10]. However, these components do not alter the valsartan quantitation since the peaks of both valsartan and the internal standard are completely resolved.

Calibration curves constructed from the peak area ratio (valsartan/internal standard) and the corresponding valsartan concentration in each calibration standard were linear from 0.01 to 1000 μg. The mean slope and intercept for the different calibration curves of valsartan in human plasma are presented in Table 1. The correlation coefficient was always greater than 0.999 during the course of the validation.

The intraday coefficient of variation ranged from 0.825 to 18.18%, while the inter-day coefficient of variation ranged from 0.969 to 13.20% indicating that the method is precise. The accuracy of the method was proven since the intra-day accuracy was in the range from 99 to 111% and the inter-day accuracy was in the range of 98.52 to 106% during the entire range of the calibration curves.

The results of stability of the drug at room temperature and in frozen plasma showed that valsartan is stable. In fact, the difference in the drug concentrations in the two analyses was always less than 10%. There was no trend in the changes in the drug concentration when the samples were kept at room temperature for 24 h, indicating the stability of valsartan in the plasma samples at room temperature. In frozen plasma, the difference in the drug concentration in all samples in the two analyses was always less than 10% in each sample. There was no trend in the change in the drug concentration in frozen plasma stored at −20°C during the study period, indicating the stability of valsartan in frozen plasma.

The limit of quantification of valsartan in this assay was 0.01 μg/mL. The retention time of the drug in the standard and the study samples were identical. There were no peaks for endogenous compounds that appeared at the same retention time for valsartan in the chromatograms for six different blank plasma samples.

The results of incurred sample reanalysis demonstrate that for each analyte the difference between the original and reanalyzed values was within 15% of the total samples reanalyzed. This confirms that the analytical procedure is valid and reproducible.

Several HPLC methods for the determination of valsartan in human plasma were reported in the literature. Most of these methods employ a liquid extraction procedure with protein precipitation (mostly with methanol), reversed-phase C_18_ column separation followed by fluorometric [11–15] or ultraviolet detection [16, 17]. Likewise, the method described in this report uses a liquid extraction step followed by fluorescence detection. Our method provides results comparable to those obtained using flurometric detection and superior to those utilizing UV detection. However, our method possesses a number of advantages. With the chromatographic conditions described, it was possible to completely resolve valsartan and the internal standard (etodolac) peaks within 7 min. The quantitative range of the assay (0.01–1 μg/mL) is comparable to that obtained by Daneshtalab *et al.* [11] without the additional time required for the back-extraction step used by the authors. The method allows accurate quantification of valsartan concentrations that are expected after regular dose administration. Overall, our method provides a rapid, sensitive, and economically-convenient procedure for the analysis of valsartan in human plasma samples encountered in pharmacokinetics studies.

### Results of pharmacokinetic study

**Fig. 3.**
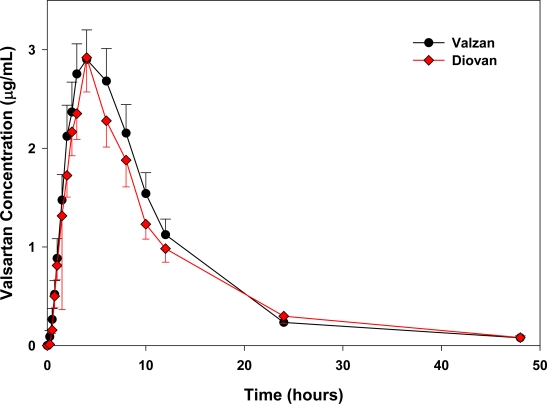
Mean plasma concentration of valsartan (± SD) of 24 volunteers versus time after a single oral dose administration of test tablet (160 mg valsartan, Pharmacare) or reference tablet (160 mg valsartan, Novartis).

The alternative HPLC-fluorescence method described and used here for valsartan quantification provides adequate sensitivity and high sample throughput required for pharmacokinetic studies. Both valsartan immediate release tablets (Valzan^®^ & Diovan^®^ 160 mg tablet) were well tolerated at the administered dose by all the subjects and no unexpected incidents occurred that could have influenced the outcome of the study. All the volunteers discharged from the hospital after the studies were in good health. Both the medications were readily absorbed from the GI tract and it was possible to quantify the drug at the first sampling time in all the volunteers. Figure 3 shows mean valsartan plasma concentrations as a function of time after the oral administration of 160 mg/tablet valsartan of both brands over the 48 h truncated sampling period.

Descriptive statistics of the major mean pharmacokinetic parameters AUC_0–48_, AUC_0–∞_, C_max_, T_max_, Ke and T_1/2_ for the test and reference formulations are summarized in Table 1 and 2.

The relative bioavailability of valsartan from test immediate release tablet (Valzan^®^, 160 mg valsartan, Pharmacare) compared to reference immediate release tablet (Diovan^®^, 160 mg valsartan, Novartis) was found to be 95.055%, 94.55% and 97.23% of AUC_0–48_, AUC_0–∞_ and C_max_ respectively, and the parametric 90% confidence intervals for those pharmacokinetic parameters values lie entirely within the FDA specified bioequivalent limit (80–125%). Statistical evaluation (ANOVA) of obtained data was always carried out.

As above pointed out, the main objective of bioequivalence studies is to assure the efficacy and safety of generic formulations. Therefore, two formulations of the same drug are considered to be bioequivalent and *per se* therapeutically equivalent if they exhibit a comparable extent and rate of absorption, when they are administered in the same molar dose and under similar experimental conditions [18–20]. AUC_0–∞_, C_max_ and T_max_ values were statistically analyzed for determination of bioequivalence of this generic product. Other pharmacokinetic parameters (i.e., T_1/2_ and K_e_) results were used as supporting results in this pharmacokinetic design. This study was conducted on healthy volunteers according to the study protocol. All subjects received the same dosages of medication, i.e one single dose of test formulation in the form of the test product or one single dose of reference in the form of the reference product with a 14 days washout period. No adverse events necessitating subject withdrawal from the study were reported. Statistical comparison of the main pharmacokinetic parameters, AUC_0–48_, AUC_0–∞_, C_max_ and T_max_ clearly indicated no significant difference between test and reference 160mg/tablet, in any of the calculated pharmacokinetic parameters. The obtained values were in good agreement with the FDA requirements for bioequivalence of generic drugs [20]. Since the AUC_0-∞_ and C_max_ mean ratios are within the 80%–125% interval, it was concluded that the tested Valzan^®^ tablet (160 mg valsartan) elaborated by Pharmacare, Palestine, is bioequivalent for both extent and rate of absorption to the commercial Diovan^®^ tablet (160mg valsartan) manufactured by Novartis Pharma AG, Basile, Switzerland/Suiza.

## Conclusion

The validated HPLC method employed here proved to be simple, fast, reliable, selective and sensitive enough to be used in clinical pharmacokinetic studies of valsartan in humans. The statistical analysis of the results of AUC_0–48_, AUC_0–∞_ and C_max_ using the ANOVA method showed that both test immediate release tablet Valzan^®^ (160 mg valsartan, Pharmacare) and reference immediate release tablet Diovan^®^ (160 mg valsartan, Novartis) are bioequivalent, since they deliver equivalent amounts of valsartan to the systemic circulation at equivalent rates for both AUC_0–48h_ and C_max_ ratios within the 80–125% interval proposed by Food and Drug Administration [20]. These results demonstrate that the formulation of this new generic tablet is good, an essential condition not only to achieve good therapeutic benefits but also to avoid any adverse effect due to bad formulation. Furthermore, these results are of great importance for further studies aimed towards the bioclassification of valsartan, since the excipients and preparation method of Diovan^®^ tablets are reported.

## Materials and Methods

### Volunteers and clinical protocol

The study protocol and the informed consent forms were approved by the Ethical Committee of Tanta University Hospital (Tanta, Egypt). The whole study, which meets the requirements of the declarations of Helsinki, was conducted in accordance with the current Good Clinical Practice (GPC), International Conference Harmonization (ICH) as well as Good Laboratory Practice (GLP) Guidelines [21, 22].

Twenty-four young adult male volunteers, non smokers, aged between 18–28 years, weighing between 67 and 83 Kg with a mean value of 76.25 ± 5.64Kg, were chosen to participate in the present study. The volunteers were not on concomitant medications and they were free from significant cardiac, hepatic, renal, pulmonary, gastrointestinal, neurological or hematological disease as determined within four weeks prior to the beginning of the study by way of medical histories and physical examinations. Subject’s health status was determined following a physical examination, laboratory tests and medical history by a qualified registered MD physician. The physician reviewed all preclinical laboratory tests for each subject. Tests included the following: (i) physical examination: height, weight, blood pressure, heart rate, body temperature and respiratory rate; (ii) blood chemistry: glucose, uric acid, BUN, creatinine AST, ALT, cholesterol and triglycerides; (iii) hematological tests: hemoglobin, hematocrit, ESR, WBCs with differential, RBCs with platelets count and morphology, lymphocytes, MCV, MCH, monocytes, neutrophils, eosinophils, basophils, HIV and Hepatitis B screen; (iv) urine analysis: specific gravity, pH and microscopic examination. These clinical tests were performed in order to determine if they fitted with the participation in the study. Exclusion criteria included extreme weight ranges (overweight or underweight), anemia, liver or renal dysfunction, parasitic and other diseases or conditions that was judged to affect the absorption, distribution and/or elimination of valsartan. All volunteers were given a written informed consent, which explained the nature of the study. All volunteers were interested and willing to participate in the study. The subjects were asked to abstain from taking drugs and alcohol for at least 3 days prior to the study and throughout the study period. On the night before starting the study, the volunteers were instructed to fast for at least 10 h before drug administration. The study had an open randomized two-period crossover design with a 14-day washout period between doses. The volunteers were arbitrarily divided into two equal groups each of 12 subjects. To the first group the reference formulation was given and to the second group the test formulation was given with a crossover after a washout period of two weeks. On the morning of the experiment, a blood sample was withdrawn from each volunteer to serve as a blank for the drug assay. Each of the 24 volunteers then took one Valzan^®^ 160 mg per tablet as the test drug, or one Diovan^®^160 mg per tablet as the reference drug followed by 240 ml of water. Blood samples for plasma drug assay were collected from an indwelling catheter inserted in the antecubital vein of one of the arms. Samples were obtained at 0.0, 0.25, 0.5, 0.75, 1, 1.5, 2, 2.5, 3, 4, 6, 8, 10, 12, 24 and 48 h after drug administration, in heparinized tubes. Plasma was directly separated by centrifugation at 3000 rpm for 10 min, removed out and stored at −20°C until assayed. Four hours after drug administration, the subjects were allowed to eat a standard breakfast of bread, jam, potato, low-fat white cheese and water (150 ml). They were then allowed controlled access to water and other non-alcoholic beverages. The volunteers had their second meal (standard lunch containing grilled chicken, rice and vegetables) 4 h later. It should be noted that valsartan is rapidly absorbed from gastrointestinal tract after oral administration and can be administered without regard to food intake. The peak effect of valsartan is evident in 2–4 h; the bioavailability is 25% [23]. The maximum plasma concentrations after single oral dose of valsartan (160 mg) reached 4 μg/mL [8].

### Drugs and chemicals

Acetonitrile (ACN) and methanol (MeOH) were purchased from Merck, (Darmstadt, Germany); ammonium dihydrogen ortho phosphate was purchased from Riedel-de Haën (Seelze, Germany); acetic acid and diethyl ether (ADS, France), Hexan sulfonic acid sodium (PXPARK Scientific Limited, UK) and double distilled high purity water was used. Human plasma was harvested from donors. The HPLC grade solvents ACN and MeOH were used as received. All other reagents were analytical grade. Valsartan (batch number VS0060307, expiration date 11/2013) and etodolac (batch number CAS 1938 internal standard) were supplied by Pharmacare, Chemical & Cosmetics, Ramallah, Palestine). All experiments were carried out before April 2009.

### Formulations

The following test formulation was employed: Valzan^®^ tablet (160 mg valsartan /tablet) from Pharmacare Chemical & Cosmetics, Ramallah, Palestine, (batch number RD-09B09; Expiry date 02/2011). The reference formulation was Diovan^®^ tablet (160 mg valsartan/tablet) manufactured by Novartis Pharma AG, Basile, Switzerland/Suiza, (batch number 21234; Expiry date 11/7/2011). Valzan^®^ was prepared according to a dry granulation method (roll compaction). Tablet formulation contained the following inactive ingredients: lactose, avicel, HPMC, aerosol, magnesium stearate, plasdone, talc, yellow iron oxide, red iron oxide, and titanium dioxide. The in-vitro release of the valsartan from Valzan^®^ was within the acceptable level reported by the profile for valsartan. The release of Diovan and Valzan were more than 85% after 15 min, this means that no need to perform the similarity and dissimilarity factors (F2 or F1). The test was carried out according to the USP 30 specifications. The tablets were placed in the vessels of the dissolution apparatus containing 1000 mL of a phosphate buffer with pH 6.8. Rotation was set on 100 rpm using USP apparatus I (Basket type) “Vailidata” hanson Research SR6. Temperature was maintained at 37°C during the time of dissolution test of 120 min. The tablets were placed in the vessels. Samples (5 ml) were taken at 1, 2, 3, 4, 5, 10, 15, 20, 30, 45, 60, 90 and 120 min. These samples were filtered through a Millipore filter 0.45μm before dilution with phosphate buffer. 25 μL of sample solutions were injected in the HPLC.

### Instruments and chromatographic conditions

The HPLC system Model LC-10 VP, (Shimadzu Scientific Instruments) was employed. Separation was accomplished with a 250 mm X 4.6 mm, 5μm Hypersil^®^ BDS C_18_ column. The mobile phase involved a mixture of 20 mM ammonium dihydrogen phosphate buffer (pH adjusted to 3.2 with phosphoric acid): acetonitrile (40:60, v/v), pumped at a flow rate of 1.7 mL/min. RF-10A XL Shimadzu fluorescence detector was set at 255 nm for excitation and 370 nm for emission. The peak quantification of valsartan was obtained by plotting valsartan to internal standard peak area ratios as a function of concentration. Areas were calculated utilizing the data analysis program Class-VP (Shimadzu Scientific Instruments).

### Standard solutions

Stock solution of valsartan (100 μg/mL) was prepared by dissolving 10 mg of valsartan in 100 mL methanol. Working standard solutions were prepared from the stock solution by sequential dilution with methanol to prepare ten working solutions of valsartan with concentrations of 100, 50, 20, 10, 5.0, 2.0, 1.0, 0.5, 0.2, 0.1 μg/mL. Stock solution of etodolac (internal standard) was prepared by dissolving 2.0 mg etodolac powder in 100 mL MeOH (20 μg/mL). Twenty- five (25) μL from each working standard solution, and the internal standard solution were transferred to a set of clean test tubes, 25 μL of blank plasma was added after evaporation and vortexed. Then treated with 0.5 mL of methanol and shaked for 30 sec, then centrifuged at 3000 rpm for 7 min. The supernatant was transferred to clean HPLC vials and 25 μL were injected into the HPLC column. Stock and working standard solutions were protected from light and stored at −20°C until use.

### Preparation of calibration standards

The calibration standards were prepared by adding known amounts (25 µL) from each working solution, and 25 μL of the internal standard solution to a set of clean test tubes. After evaporation of the methanolic solution, 0.25 mL of blank plasma was added to each tube to form a set of calibration standards with concentrations of 10, 5, 2, 1, 0.5, 0.2, 0.1, 0.05, 0.02, 0.01 μg/ mL.

### Sample preparations for HPLC injection

25 μL of each study sample was transferred to a clean test tube. The study samples were treated as the calibration standards after addition of the internal standard.

### Validation Procedures

The validation of this chromatographic analytical method was performed in order to evaluate its linearity, selectivity, stability, precision and accuracy.

Calibration curves were constructed from the peak area ratio (drug/ internal standard) and the corresponding valsartan concentration in each calibration standard. The linearity study was carried out in the concentration range of 0.01 to 10 μg/mL. To assess linearity, drug free plasma was spiked with known amounts of the drug to achieve the concentration of 0.01, 0.02, 0.05, 0.1, 0.2, 0.5, 1.0, 2.0, 5.0, and 10 μg/mL.

Precision was determined as the coefficient of variation (%CV), and the accuracy as the percentage relative error (RE) of a series of measurements. Precision and accuracy data were obtained by analyzing aliquots of three spiked plasma samples at low middle and high concentration levels of valsartan. Intra-day reproducibility was determined by analyzing three replicates of calibration curves on the same day, and inter-day reproducibility was evaluated by the analysis of six different calibration curves on six different days during the study period.

The stability of the drug at room temperature was determined by preparing three different plasma samples for each drug concentration and then injecting the samples immediately into the HPLC system. The samples were kept at room temperature and were injected again after 24 h. The concentrations measured at time zero and after 24 h were compared to determine whether there were changes in the concentrations with time.

The stability of the drug in frozen plasma was investigated by the analysis of the study samples obtained from three volunteers twice. The first analysis was performed at the beginning of the study and the second analysis was performed at the end of the study. The samples were stored at −20°C between the analyses. The low limit of quantification (LLOQ) is defined as the lowest concentration of analyte that can be determined with acceptable precision and accuracy under the stated experimental conditions. The LLOQ was estimated by analyzing samples with known amounts of valsartan, at progressively lower concentrations, starting at the lower end of the calibration curves. The performance of the assay during the analysis of the study samples was evaluated by the analysis of the quality control (QC) samples. The QC samples were prepared by spiking blank plasma with valsartan before the beginning of the study sample analysis. The QC samples were prepared to have low, medium and high valsartan concentrations (0.05, 1.0 and 5.0 μg/mL). Three QC samples were incorporated with each analysis run and were treated as unknown samples. The concentration in each QC sample was determined from the calibration curve and the calculated concentration was compared with the nominal concentration. The analysis run was accepted if at least 2 out of 3 QC samples were within 20% of nominal concentration [24].

An incurred sample reanalysis (ISR) was performed on 72 sample from all study subjects. Three sample points for each study period were selected and reanalyzed to test the reproducibility of the assay [25].

### Pharmacokinetic and statistical analysis

The maximum valsartan plasma concentration (C_max_) and the corresponding time of peak plasma concentration (T_max_) were taken directly from the slope of the semi-logarithmic plot of the terminal phase of the plasma concentration-time curve calculated by linear regression. The elimination half–life (T_1/2_) was derived by dividing ln_2_ by the elimination rate constant Ke. The areas under the valsartan plasma concentrations time curves from (AUC_0–48_) and the area to the infinity (AUC_0–∞_) were calculated by using the linear trapezoidal method. Extrapolation to the infinity was done by adding the value Ct/Ke to the calculated AUC_0–48_ (where Ct is the last detectable concentration of valsartan). For the purpose of bioequivalence analysis, one way analysis of variance (ANOVA procedure) was used to assess the effect of formulations, periods, sequences and subjects on AUC_0–48_, AUC_0–∞_, and C_max_.

## Figures and Tables

**Tab. 1.** Mean slope and intercept for the different calibration curves of valsartan in human plasma.

**Calibration Curve Parameter**	**Mean ± SD**
Slope	1.510 ± 0.031
Intercept	0.029 ± 0.011
Correlation coefficient	0.999

**Tab. 2.** Pharmacokinetic parameters calculated for valsartan after a single oral dose administration of test tablet (160 mg valsartan, Pharmacare) to 24 healthy male volunteers.

**Statistical Parameter**	**C_max_ (μg/mL)**	**T_max_ (h)**	**K_e_ (h^−1^)**	**T_½_ (h)**	**AUC_0–t_ (μg.h/mL)**	**AUC_t-∞_ (μg.h/mL)**	**Total AUC_0–∞_ (μg.h/mL)**
Mean	2.141	4.750	0.085	9.550	21.53	1.03	22.56
S.D.	0.567	1.790	0.042	3.150	6.950	0.45	7.10
S.E.	0.116	0.370	0.009	0.640	1.420	0.09	1.450
Min	1.399	2.500	0.048	3.480	8.720	0.25	9.450
Max	4.114	10.00	0.199	14.41	41.80	1.89	42.68

**Tab. 3.** Pharmacokinetic parameters calculated for valsartan after a single oral dose administration of reference tablet (160 mg valsartan, Novartis) to 24 healthy male volunteers.

**Statistical Parameter**	**C_max_ (μg/mL)**	**T_max_ (h)**	**K_e_ (h^−1^)**	**T_½_ (h)**	**AUC_0–t_ (μg.h/mL)**	**AUC_t-∞_ (μg.h/mL)**	**Total AUC_0–∞_ (μg.h/mL)**
Mean	2.208	4.02	0.085	9.510	22.65	1.210	23.86
S.D.	0.663	1.44	0.044	3.030	7.860	0.530	8.040
S.E.	0.135	0.29	0.009	0.620	1.600	0.110	1.640
Min	0.879	2.00	0.049	3.570	6.750	0.270	7.500
Max	3.894	8.00	0.194	14.15	41.02	2.26	42.22
